# A two-stage framework for optical coherence tomography angiography image quality improvement

**DOI:** 10.3389/fmed.2023.1061357

**Published:** 2023-01-23

**Authors:** Juan Cao, Zihao Xu, Mengjia Xu, Yuhui Ma, Yitian Zhao

**Affiliations:** ^1^School of Information Science and Engineering, Chongqing Jiaotong University, Chongqing, China; ^2^Cixi Institute of Biomedical Engineering, Ningbo Institute of Materials Technology and Engineering, Chinese Academy of Sciences, Ningbo, China; ^3^Affiliated Cixi Hospital, Wenzhou Medical University, Ningbo, China

**Keywords:** OCTA, stripe removal, image enhancement, generative adversarial networks, two-stage framework

## Abstract

**Introduction:**

Optical Coherence Tomography Angiography (OCTA) is a new non-invasive imaging modality that gains increasing popularity for the observation of the microvasculatures in the retina and the conjunctiva, assisting clinical diagnosis and treatment planning. However, poor imaging quality, such as stripe artifacts and low contrast, is common in the acquired OCTA and in particular Anterior Segment OCTA (AS-OCTA) due to eye microtremor and poor illumination conditions. These issues lead to incomplete vasculature maps that in turn makes it hard to make accurate interpretation and subsequent diagnosis.

**Methods:**

In this work, we propose a two-stage framework that comprises a de-striping stage and a re-enhancing stage, with aims to remove stripe noise and to enhance blood vessel structure from the background. We introduce a new de-striping objective function in a Stripe Removal Net (SR-Net) to suppress the stripe noise in the original image. The vasculatures in acquired AS-OCTA images usually exhibit poor contrast, so we use a Perceptual Structure Generative Adversarial Network (PS-GAN) to enhance the de-striped AS-OCTA image in the re-enhancing stage, which combined cyclic perceptual loss with structure loss to achieve further image quality improvement.

**Results and discussion:**

To evaluate the effectiveness of the proposed method, we apply the proposed framework to two synthetic OCTA datasets and a real AS-OCTA dataset. Our results show that the proposed framework yields a promising enhancement performance, which enables both conventional and deep learning-based vessel segmentation methods to produce improved results after enhancement of both retina and AS-OCTA modalities.

## 1. Introduction

Medical images with clean presentation, adequate contrast and informative details are essential in medical image analysis for clinical applications: e.g., tissue segmentation, and disease diagnosis. However, stripe artifacts or poor contrast often occur during the medical image acquisition process (1). The accuracy of a computer-aided diagnosis system is highly dependent on the quality of pre-processing as errors can be propagated and accumulated due to poor imaging quality (2).

**Figure 1:**
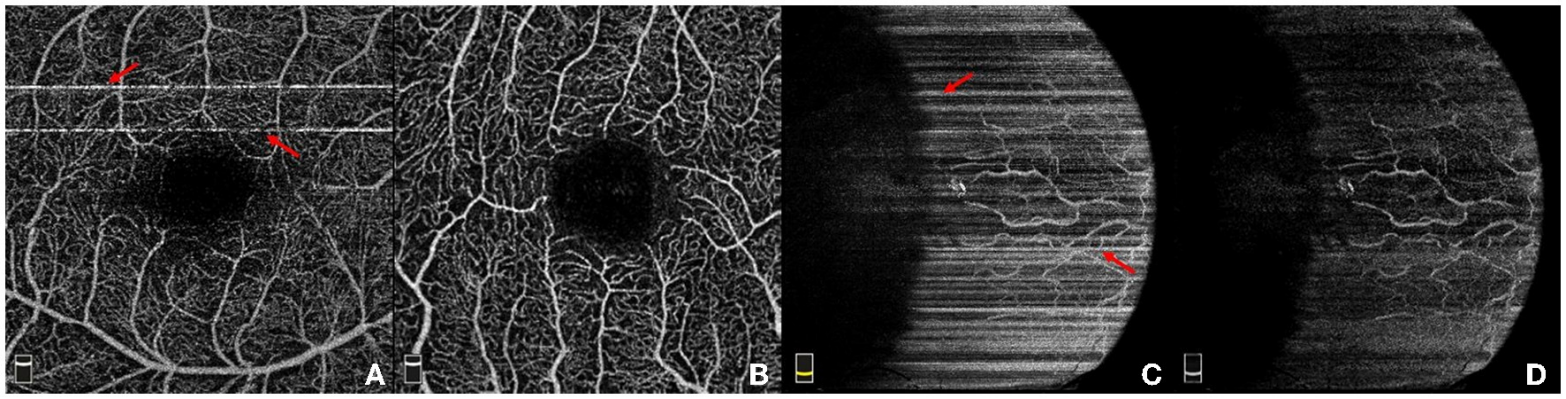
Illustration of four example OCTA images. **(A)** A retinal OCTA image with stripe noise; **(B)** Another retinal OCTA image without stripe noise; **(C)** A conjunctival AS-OCTA image with stripe noise; **(D)** The conjunctival AS-OCTA image in **(C)** with stripe noise removed.

As a functional extension of optical coherence tomography (OCT), OCT Angiography (OCTA) is a new emerging non-invasive imaging modality that enables observation of microvasculatures up to capillary level (3, 4). Figure 1B demonstrates one high-quality retinal OCTA image sample. OCTA will reveal morphological changes of retinal vessels associated with a wide range of retinal diseases and has shown its potential clinical applications in facilitating monitoring and diagnosis of glaucoma (5), diabetic retinopathy (6), artery and vein occlusions (7), and age-related macular degeneration (AMD) (8), to name only the most widely occurring ones.

Recently, OCTA has also been adopted to image the vessels in the anterior segment of the eye. Current commercial OCTA systems are not specifically designed for the anterior segment (9), the use of OCTA in assessments of the anterior segment has not been fully explored. The AS-OCTA technique has been used to quantify the vascular density and diameter in the cornea (10–13), conjunctiva (14), and iris (15), in order to seek better treatment options. Figures 1C, D illustrates one case of AS-OCTA imaging by scanning conjunctival region. Overall, OCTA technique opens up a new avenue to study the relation between ocular vessels and various eye and neurodegenerative diseases (4).

OCTA has the ability to produce three-dimensional (3D) images of the ocular vasculature at different depths, and the acquired 3D data is always mapped into two-dimensional (2D) *en face* image by using the maximum projection for the ease of visualization. However, acquisition of OCTA and AS-OCTA usually takes several seconds, e.g., 3–5 s by RTVue XR Avanti SD-OCT system (Optovue, Inc, Fremont, California, USA), and in consequence, OCTA images are inevitably susceptible to motion artifacts caused by involuntary eye movements. Adjacent OCT-scans present a variety of decorrelation and further degrades the image quality: motions like microsaccade leads to a momentary change in the location of the scan and produce visible horizontal or vertical white stripe artifacts in the *en face* images (16), as shown by red arrows in Figures 1A, C. These stripe artifacts lead to unpleasing visualization, inaccurate vessel quantification, and even hinder clinical decision making. Accelerating acquisition speed may mitigate motion artifacts, relatively low spatial sampling rate is often used in some devices, e.g., Topcon-DRI-OCT-1 machine (Topcon Corporation, Japan). Unfortunately, such accelerating process requires more complex design of the imaging systems (17), and may also lead to the presence of sample-based speckle and non-existent vessels. Other commercial OCTA imaging systems, e.g., RTVue XR Avanti SD-OCT system (Optovue, Inc, Fremont, California, USA), have the built-in motion detection and correction functions to suppress stripe artifacts. Nevertheless, there still exist slight stripe artifacts in the form of residual lines. Furthermore, these functions could increase scanning time or even cause imaging failures if patients are unable to hold their eyes still (18). In addition to stripe noise, low contrast or intensity inhomogeneity caused by poor illumination conditions usually leads to hardly visible or even discontinuous vasculatures. Figure 1D demonstrates the AS-OCTA image in Figure 1C after stripe noise removal, but the inherent poor contrast will still pose significant challenges to subsequent medical image analysis tasks, such as blood vessel segmentation (2) and disease/lesion detection (19).

As an alternative, it is crucial to design high-quality enhancement methods that are able to remove stripe artifacts and enhance image quality simultaneously, so as to enhance those details obscured in the originals. Nevertheless, it has been proved very difficult to design a single method that will work across a variety of different medical imaging modalities (20). For OCTA imaging modality, there exist two specific challenges in imaging quality improvement. On one hand, compared with other imaging modalities such as hyperspectral imagery, stripe artifacts in OCTA images always have more diversified characteristics with larger differences in intensity, length, thickness and position, and are easily confused with the vascular structures. Recently, there appears several deep learning based approaches for image de-striping, but few models combine with prior knowledge of stripe artifacts to further improve image de-striping performance. On the other hand, for poor contrast or intensity inhomogeneity in OCTA images, it is difficult to obtain such aligned low/high-quality image pairs for supervised learning. However, most existing unpaired learning frameworks have relatively insufficient constraints on local details, which is unfavorable to restore microvasculartures with poor contrast in OCTA images.

Thus in this paper, we propose a novel two-stage framework for OCTA (including both posterior and anterior segment) image enhancement. The proposed framework consists of the de-striping stage and the re-enhancing stage, with the aim to respectively remove stripe artifacts and improve the contrast in OCTA images. This paper makes the following main contributions:

- In the de-striping stage, we propose a U-shape network called Stripe Removal Net (SR-Net), which introduces a novel de-stripe loss containing low-rank prior of stripe artifacts and constraints on the vascular structure. To our best knowledge, this is the first time to introduce constraints on stripe artifacts in the objective function of a deep learning network.
- In the re-enhancing stage, we propose a novel generative adversarial network called Perceptual Structure GAN (PS-GAN), which integrates cyclic perceptual loss and structure loss into a bi-directional GAN like CycleGAN. By constraints on the vascular structure at different feature levels, both thick and thin vessels in low-contrast OCTA images can be further enhanced.
- The proposed method has undergone rigorous qualitative and quantitative evaluation using three datasets including OCTA and AS-OCTA imagery. For each medical image modality, we employ different image quality assessment schemes, and the experimental results demonstrate the superiority of the proposed framework.

The remainder of the paper is organized as follows. We review the related works to the proposed method in Section 2. The methodology of the proposed method is presented in Section 3. To validate the de-striping effectiveness and image quality improvement of the proposed method, we conduct extensive experiments in Sections 4, 5. We discuss the details of the proposed method and draw our conclusion in Section 6.

## 2. Related works

### 2.1. Stripe noise removal

Undesirable noise artifacts always exists in different medical imaging modalities, such as optical coherence tomography (OCT), computed tomography (CT), Ultrasound, magnetic resonance (MR) and positron emission tomography (PET). In the past decades, numerous methods have been proposed for medical image denoising. Conventional denoising approaches can be roughly divided into the following several categories: filtering-based methods (21) including NLM (22) and BM3D (23), transform-based methods such as wavelets (24), shearlets (25) and curvelets (26), and optimization-based algorithms including sparse representation (27, 28), low-rank decomposition (29, 30) and total variation (31, 32). Recently, there have appeared several methods based on convolutional neural networks. Ma et al. (33) designed an edge-sensitive conditional generative adversarial network for speckle noise reduction in OCT images. Chen et al. (34) proposed a Residual Encoder-Decoder CNN to remove noise from low-dose CT images. Jiang et al. (35) developed a Multi-channel DnCNN (MCDnCNN) with two training strategies to denoise MR images. Cui et al. (36) proposed an unsupervised deep learning approaches for PET image denoising.

As a kind of noise artifacts, stripe noise usually exists in some imaging modalities such as remote sensing image, microscopy and X-ray. Different from other noise artifacts such as speckle noise, stripe noise usually appears as several parallel lines randomly distributed through the whole image, which brings additional challenges for image interpretation.

In recent decades, several researches have investigated image de-striping. Conventional de-striping methods can be categorized into three types: filtering-based, optimization-based and deep learning-based. Filtering-based methods usually utilize Fourier transform (37, 38) or linear-phase (39) to remove the stripe or speckle artifacts and then reconstruct a noise-free image. They are relatively straightforward to implement and fast to process images, but they generally perform well only on periodic stripe noise and often cause the loss of details in the original images.

Recently, many optimization models have been proposed to remove stripe noise. Chang et al. (40) regarded the images and stripe noise as equally important information and used a low-rank-based single-image decomposition model to obtain high-quality images. He et al. (41) used TV-regularized low-rank matrix factorization to remove stripe noise in hyperspectral images. Chang et al. (42) used an Anisotropic Spectral-Spatial Total Variation (ASSTV) method to preserve edge information and details in stripe spectral images. Wu et al. (18) proposed a Cooperative Uniformity Destriping model (CUD) and a Cooperative Similarity Destriping model (CSD) to remove stripe noise from OCTA images by using the prior condition of low-rank and anisotropic TV, while the CSD model considers the association of stripes and blood vessels between different layers to remove stripe noise. However, these optimization models require complex numerical solutions to solve partial differential equations (PDEs) in an iterative manner, and thus are not applicable to real-time applications (43).

With the rapid development of deep learning, it has recently been applied for stripe artifact removal in different imaging modalities. Chang et al. (43) proposed a two-stage deep convolutional neural network (CNN) with the short-term and long-term connections, where the first stage acts as the noise subnet to guide the second stage to obtain the denoised image. He et al. (44) proposed a CNN model with residual learning modules to remove the synthetic stripe noise of infrared images, where the stripe noise images are generated from fixed pattern noise (FPN) module. Guan et al. (45) proposed a FPNR-CNN model that includes the coarse-fine convolution unit and the spatial and channel noise attention unit to remove stripe noise. However, most of existing deep learning methods for stripe removal tasks focus on exploring advanced CNN structures, such as residual learning modules, which might only perform well in specific types of medical images. In addition, few models formulated and incorporated the constraints on stripe artifact removal into their deep learning approaches. Motivated by the success of deep learning, in this work, we first propose a convolutional neural network called SR-Net for stripe noise removal in OCTA images. Different from other deep learning approaches for image destriping, the proposed SR-Net incorporates prior information of stripe distribution into its loss function for more effective learning of stripe characteristics. To our best knowledge, this is the first attempt to introduce constraints on stripe distribution in a convolutional neural network.

### 2.2. Image enhancement

Many image enhancement methods proposed in the field of computer vision have been applied to medical images, with the aim of improving image quality. Well-known examples of global enhancement methods, such as histogram equalization (HE) (46), and contrast-limited adaptive histogram equalization (CLAHE) algorithm (47), are widely-used methods in medical image enhancement. They aim to stretch the dynamic range of the input image, and adjust the intensities of pixels. However, these methods typically enhance images uniformly, irrespective of whether a given region is in the foreground or background. Guided image filtering (GIF) (48) and its accelerated version Fast Guided Filter (FGF) (49) are two promising methods proposed recently for single image enhancement. These methods have the limitation that they frequently over-smoothed regions close to flat, and in consequence struggle to preserve fine details. Several medical image enhancement models have been proposed based on the famous block matching & 3D collaborative filtering method (BM3D) (50) and its extension BM4D (51). These have been successfully adopted to improve the quality of CT, MRI, and OCT imagery (52). However, we noted that the enhanced images are often still blurred, these traditional methods usually fail to consider the global information of the image.

Recently, deep learning has provided new insights for medical image enhancement. LLNet (53) utilizes stacked sparse denoising auto-encoders trained on synthetic data to enhance and denoise low-light noisy images. MSR-Net (54) models conventional multi-scale Retinex (MSR) methods with a deep neural network. MBLLEN (55) extracts and fuses features at different levels in the network to solve the image enhancement problem. However, learning-based approaches are facing a critical challenge—it is difficult to collect a large number of medical image pairs (low- and high-quality) for training. Jiang et al. (56) proposed EnlightenGAN to enhance low-light images, which includes a global-local discriminator structure, a self-regularized perceptual loss fusion and attention mechanism. Ma et al. (20) proposed a structure and illumination constrained GAN (StillGAN), which enhances images from low-quality domain to high-quality domain through structure loss and illumination constraint. Zhao et al. (57) proposed a dynamic retinal image feature constraint in GAN for image enhancement to improve the quality of low-contrast retinal images. In this paper, we also developed a bi-directional GAN called PS-GAN as the re-enhancing stage, which incorporates cyclic perceptual loss and structure loss to constrain the enhancement model on the vascular structure at different feature levels.

## 3. Proposed method

**Figure 2:**
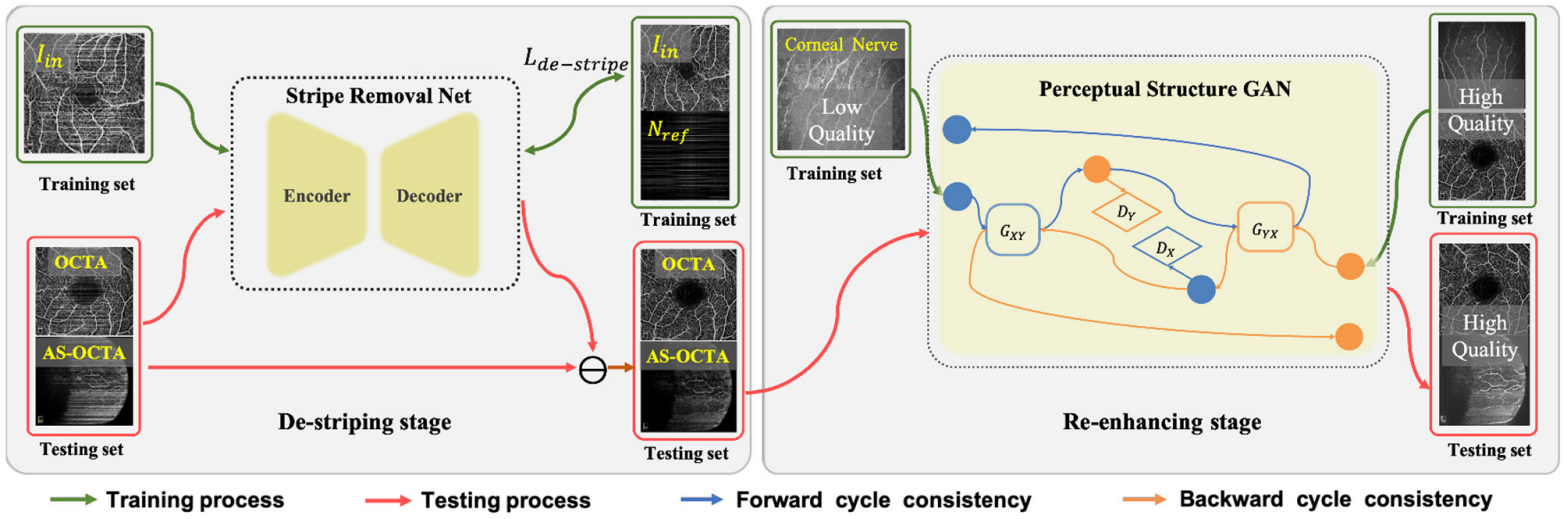
Overall framework of the proposed two-stage image enhancement method, which includes Stripe Removal Net and Perceptual Structural GAN for de-striping and image enhancement respectively.

In this section, we detail the proposed two-stage image enhancement framework which consists of a de-striping stage and a re-enhancing stage, and its overall architecture is shown in Figure 2.

### 3.1. De-striping stage: Stripe removal network

**Figure 3:**
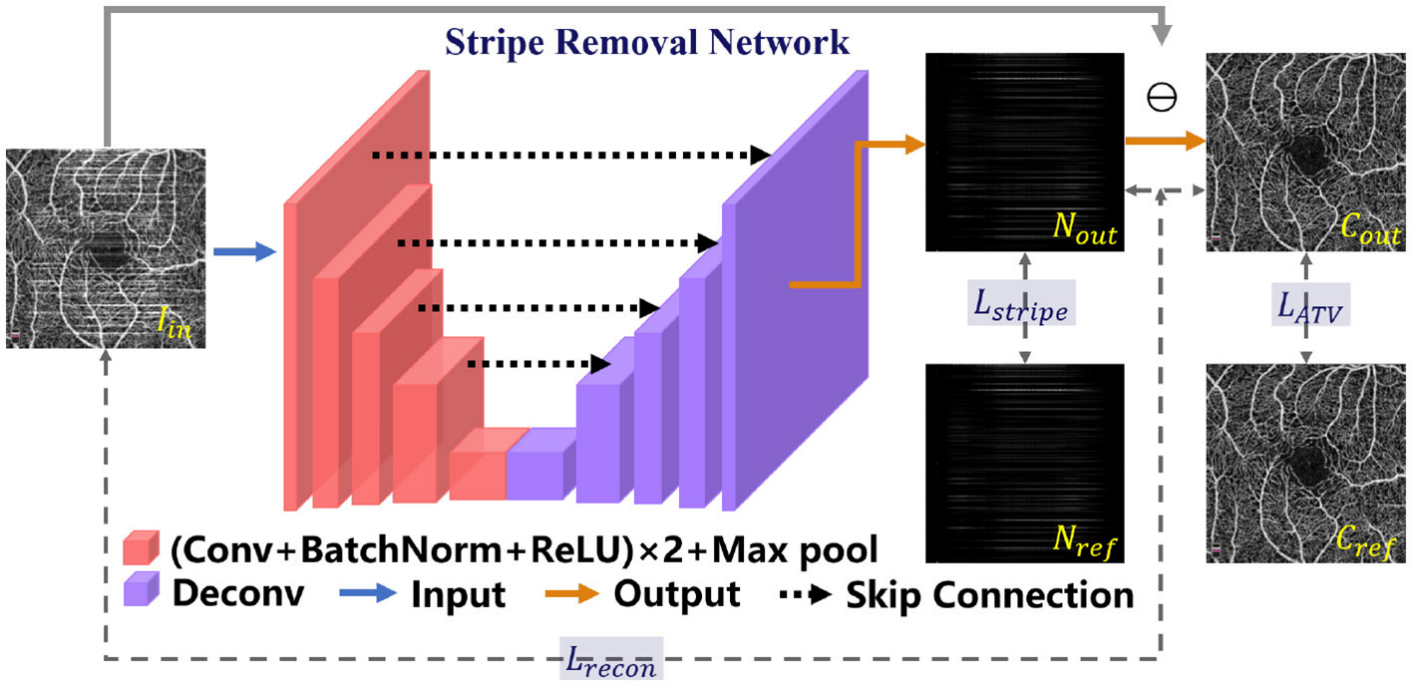
Illustration of the Stripe Removal Net (SR-Net) for the de-striping stage. The proposed objective function *L*_*destripe*_ is a combination of *L*_*recon*_, *L*_*stripe*_ and *L*_*ATV*_.

Since there exist obvious stripe noise in OCTA or AS-OCTA images, we developed a stripe removal network (SR-Net) to remove stripe noise, as illustrated in the de-striping stage of Figure 2. Our SR-Net adopts an encoder-decoder architecture with symmetric skip connections, following the general structure of U-Net (58), so that multi-scale features can be fused to produce better stripe-free results. The architecture of the proposed SR-Net is illustrated in Figure 3. In our work, we assume that a given OCTA or AS-OCTA image *I* can be decomposed into noise distribution map *N* and clean map *C* in the form of *I* = *N* + *C*. SR-Net outputs the noise map *N*_*out*_ by learning a mapping from the input image *I*_*in*_ to the reference stripe-noise image *N*_*ref*_. And we could acquire the *N*_*ref*_ by clean images and stripe noise (18).

Then the output clean image *C*_*out*_ in the form of residual between *I*_*in*_ and *N*_*out*_ can be obtained: *C*_*out*_ = max{*I*_*in*_ − *N*_*out*_, 0}. For more effective constraints to the de-striping framework, we construct a de-stripe loss function $L_{destripe}$, which consists of three terms—reconstruction loss $L_{recon}$, stripe loss $L_{stripe}$ and anisotropic total variation (ATV) loss $L_{ATV}$:

(1) $$\begin{array}{l} L_{destripe}=\alpha L_{recon}+\beta L_{stripe}+\gamma L_{ATV}, \end{array}$$

where α, β and γ are the positive weights to balance these terms respectively.

#### 3.1.1. Reconstruction loss

In order to constrain the dependence of spatial distribution between the generated clean and noise distribution image, we introduce a reconstruction loss. Based on the assumption that the original image can be constructed by the output clean image and noise image, the reconstruction loss $L_{recon}$ is defined as:

(2) $$\begin{array}{l} L_{recon}=\frac{1}{2}||I_{in}-\left(N_{out}+C_{out}\right)|{|}_{F}, \end{array}$$

where ||·||_*F*_ represents the Frobenius norm, and $L_{recon}$ enables the output noise distribution and clean image, respectively to share the consistency in spatial distribution with the input image.

#### 3.1.2. Stripe loss

Wu et al. (18) confirmed the existence of low-rank prior in stripe noise. According to the low-rank characteristic of stripe noise, the number of singular values of stripe-noise images should be approximated to zero after singular value decomposition. In order to keep the consistence between the generated stripe-noise map and the reference stripe-noise image of a synthesized image, we introduce a new stripe loss $L_{stripe}$:

(3) $$\begin{array}{l} L_{stripe}=||Stripe\left(N_{ref}\right)-Stripe\left(N_{out}\right)|{|}_{F}, \end{array}$$

where *N*_*ref*_ represents the reference stripe-noise image.*Stripe*(·) is the stripe degradation function aimed at reconstructing the primary stripe component *via* a soft-thresholding operation on singular values, which is denoted as: *Stripe*(*N*) = *U* · *shrink*(*S*) · *V*^*T*^, where *N* = *U* · *S* · *V*^*T*^ is singular value decomposition on *N*. *shrink*(·) is the soft-thresholding operation aimed at selecting non-zero singular values on *S*:

(4) $$\begin{array}{l} shrink\left(S\right)=\text{diag}\left\{\max\left(S_{ii}-\lambda ,0\right)\right\}, \end{array}$$

where *S*_*ii*_ represents the diagonal element of singular value matrix *S*, and λ is a small positive constant for selecting singular values.

#### 3.1.3. ATV loss

Vessel structures are the most significant biomarkers in OCTA or AS-OCTA images for clinical diagnosis, which are prone to be corrupted during the de-striping process. To this end, ATV loss is introduced to maintain the completeness of vessel structures. ATV is able to measure the edge sharpness of an image (42) and has been widely applied in image restoration with edge preservation. The proposed ATV loss constrains the edge of two images and is defined as follows:

(5) $$\begin{array}{l} L_{ATV}=||ATV\left(C_{ref}\right)-ATV\left(C_{out}\right)|{|}_{F}, \end{array}$$

where *C*_*ref*_ represents the reference clean image; *ATV*(·) is the function to extract gradient information defined as:

(6) $$\begin{array}{l} ATV\left(C\right)=||{▽}_{u_{x}}C|{|}_{1}+||{▽}_{u_{y}}C|{|}_{1} \end{array}$$

where ||·||_1_ represents the *L*_1_ norm; ▽_*u*_*x*__ and ▽_*u*_*y*__ represent the horizontal and vertical difference operations on the image *C* respectively.

Note that the proposed SR-Net was trained on our synthetic OCTA datasets, which contain the reference stripe-noise images *N*_*ref*_, the reference clean images *C*_*ref*_ and corruption images synthesized from the both.

### 3.2. Re-enhancing stage: Perceptual structure GAN

Although the stripe noise can be effectively removed by the proposed SR-Net during the de-striping stage, it will not improve the low contrast issues between the vessels and background, which hinders clinicians from accurate identification and quantification of the vessels for informed diagnosis. To this end, we introduced a re-enhancing stage to improve the perceptual contrast of vessel structures adaptively.

**Figure 4:**
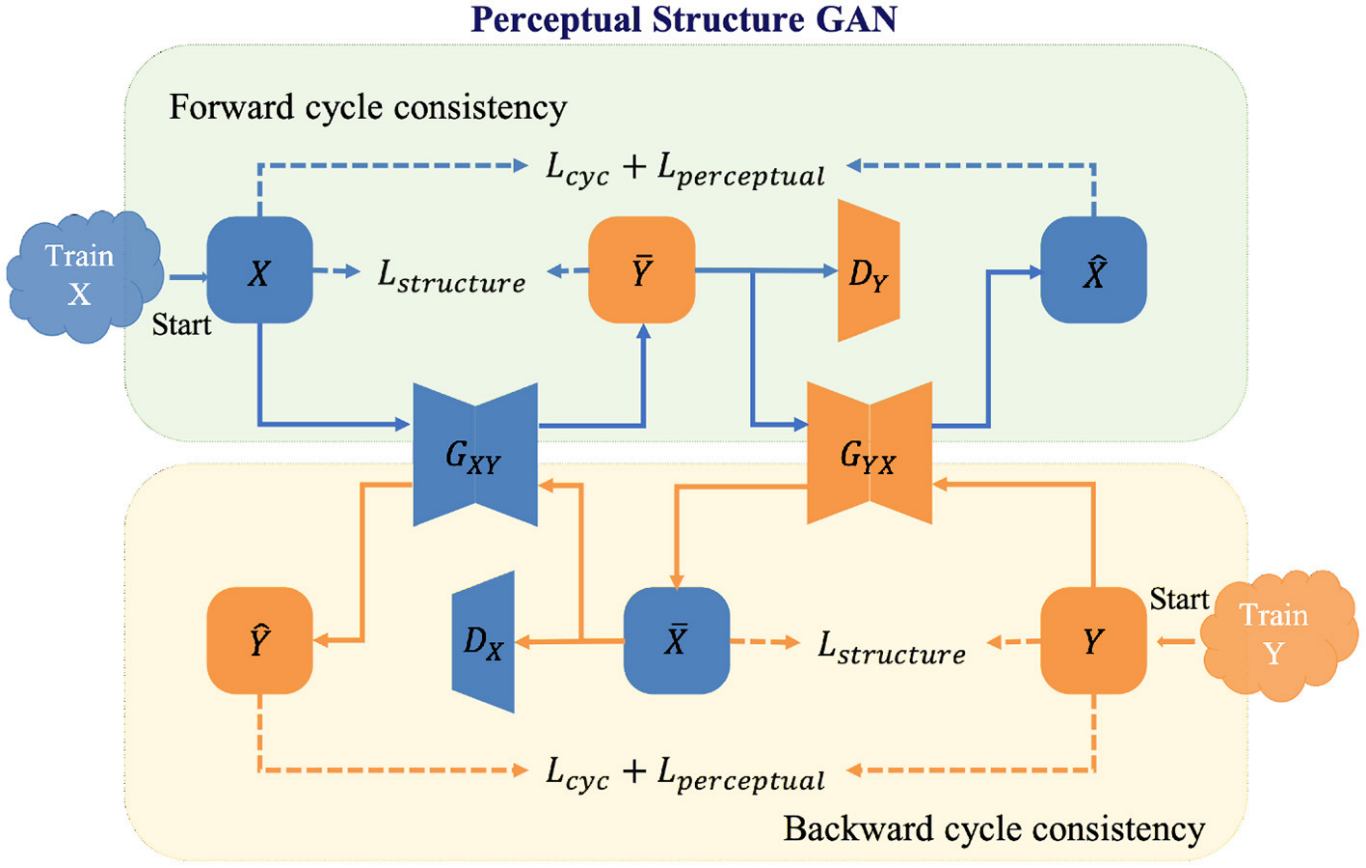
Illustration of the perceptual structure GAN (PS-GAN) for the re-enhancing stage.

In the re-enhancing stage, we proposed a novel bi-directional GAN called Perceptual Structure GAN (PS-GAN), which incorporates cyclic perceptual loss and structure loss into CycleGAN architecture (59). The architecture of the re-enhancing stage is shown in Figure 4. In this stage, low- and high-contrast images are treated as being in two different domains, and the mapping from low-contrast domain to high-contrast domain could be learned *via* PS-GAN. Our PS-GAN framework consists of two generators *G*_*XY*_, *G*_*YX*_ and two discriminators *D*_*X*_, *D*_*Y*_, where *G*_*XY*_ (*G*_*YX*_) aims to translate an image from domain *X* (*Y*) to domain *Y* (*X*), and *D*_*X*_ (*D*_*Y*_) attempts to identify whether an image is the real one from domain *X* (*Y*) or the generated one from domain *Y* (*X*).

The baseline objective function of the proposed PS-GAN includes adversarial loss, cycle-consistency loss and identity loss, following the configuration of CycleGAN. Although widely applied to unpaired image translation problems, the baseline objective function of such bi-directional GAN has several drawbacks. On one hand, cycle-consistency in the baseline objective function constrains generators at image level without capitalizing on features of different levels, which is prone to produce some unsatisfactory artifacts. On the other hand, adversarial and cycle-consistency constraints only enable generators to produce proper results in terms of global appearance, which might lead to loss of some vital structural details. To this end, cyclic perceptual loss and structure loss are integrated into our PS-GAN and they are introduced as follows.

#### 3.2.1. Cyclic perceptual loss

CycleGAN will prevent two generators from contradicting each other by converting unpaired learning into paired learning *via* constructing a self-supervisory signal. However, image-level cycle-consistency of CycleGAN is not adequate to focus on both low- and high-level features, which will lead to unsatisfactory artifacts in the enhanced image. To overcome this shortcoming, extra feature-level cycle-consistency called cyclic perceptual loss is introduced in the objective function. Different from image-level cycle-consistency, cyclic perceptual loss calculates cycle-consistency loss based on low- and high-level features extracted from the VGG-19 (60). Cyclic perceptual loss is defined as:

(7) $$\begin{array}{l} \begin{array}{l} L_{p}\left(G_{XY},G_{YX}\right)=\underset{l=2,5}{\sum }||\phi^{l}\left(x\right)-\phi^{l}\left(G_{YX}\left(G_{XY}\left(x\right)\right)\right)|{|}_{F}^{2}\\ \;+||\phi^{l}\left(y\right)-\phi^{l}\left(G_{XY}\left(G_{YX}\left(y\right)\right)\right)|{|}_{F}^{2} \end{array} \end{array}$$

where *x* ∈ *X*, *y* ∈ *Y*, and ϕ^*l*^(·) represents the output of the *l*-*th* max pooling layer of the VGG-19 feature extractor pretrained on the ImageNet. In Equation (7), features extracted from the 2*nd* and 5*th* max pooling layer of the pretrained VGG-19 are treated as low- and high-level ones, respectively to calculate cyclic perceptual loss.

#### 3.2.2. Structure loss

In order to preserve vessel structures of the enhanced images, we utilized the structure loss (20) to maintain the invariance of vessel structures. Inspired by the structure comparison function in structural similarity (SSIM) metrics, the structure loss measures the dissimilarity of structure between the original image and its enhancement in local windows and is defined as follows:

(8) $$\begin{array}{l} \begin{array}{c} L_{s}\left(G,X\right)=E_{x\in X}\left[1-\frac{1}{M}\overset{M}{\underset{i=1}{\sum }}\frac{\sigma_{x_{i},G{\left(x\right)}_{i}}+c}{\sigma_{x_{i}}\sigma_{G{\left(x\right)}_{i}}+c}\right] \end{array}, \end{array}$$

where *M* represents the number of local windows of the input image, *x*_*i*_ and *G*(*x*)_*i*_ represent the *i*-*th* local window of an image and its generated one respectively; σ_*x*_*i*__ and σ_*G*(*x*)_*i*__ represent the standard deviations of *x*_*i*_ and *G*(*x*)_*i*_ respectively; σ_*x*_*i*_,*G*(*x*)_*i*__ represents the covariance of *x*_*i*_ and *G*(*x*)_*i*_; *c* is a small positive constant.

Finally, the loss function of PS-GAN can be expressed as:

(9) $$\begin{array}{l} \begin{array}{l} \;L_{PS}\left(G_{XY},G_{YX},D_{X},D_{Y}\right)=L_{bl}\left(G_{XY},G_{YX},D_{X},D_{Y}\right)\\ +\xi L_{p}\left(G_{XY},G_{YX}\right)+\rho_{1}L_{s}\left(G_{XY},X\right)+\rho_{2}L_{s}\left(G_{YX},Y\right) \end{array} \end{array}$$

where $L_{bl}$ represents the baseline objective function of PS-GAN; ξ, ρ_1_ and ρ_2_ are the weighted parameters of each term except for $L_{bl}$.

## 4. Experimental setup

### 4.1. Datasets

In this study, three datasets are used to validate the proposed image enhancement framework, including an OCTA dataset (PUTH), a public-accessible OCTA dataset (ROSE) (4), and our in-house AS-OCTA dataset. All the datasets used are collected under the approvals of relevant authorities and consented by the patients, following the Declaration of Helsinki.

**Figure 5:**
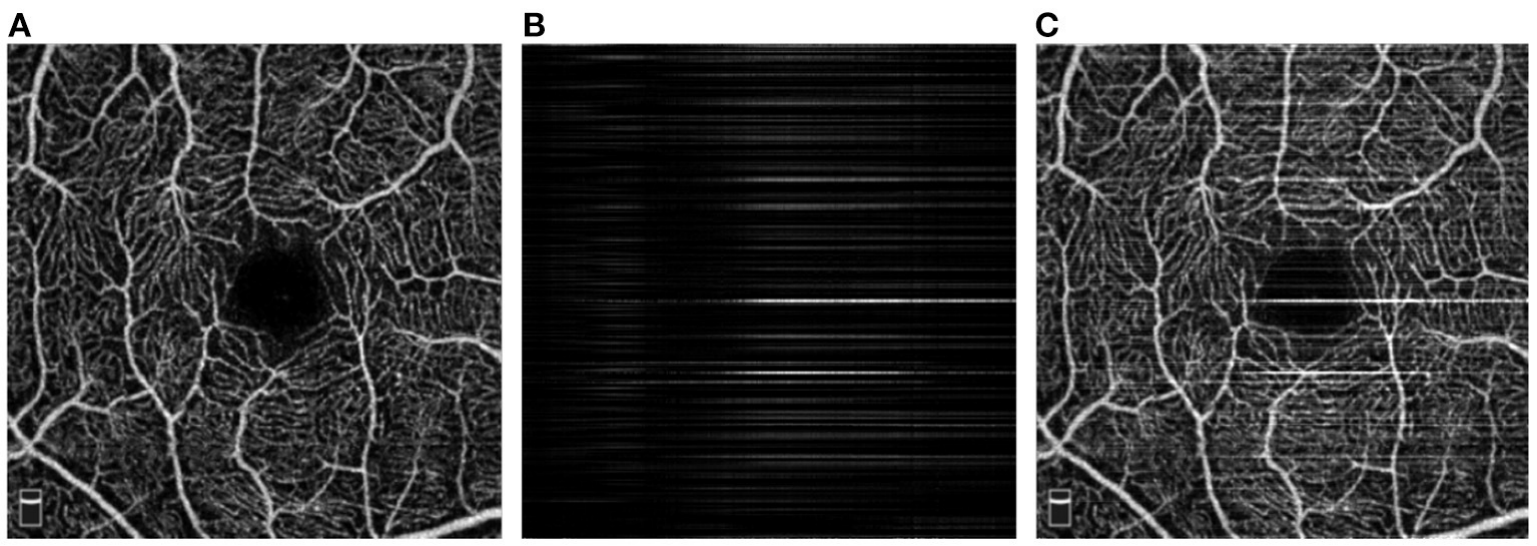
Illustration of paired OCTA images in **PUTH** dataset. **(A)** Clean image; **(B)** Stripe noise at noise level *i* = 2 generated by Wu et al. (18); **(C)** Stripe noise in **(B)** added on **(A)**.

**PUTH** is a private OCTA dataset that consists of clean and stripe noise corrupted image groups. The clean group contains 210 *en face* images from 210 eyes (including 47 with Alzheimer's disease (AD), 29 with neuromyelitis optica (NMO), 29 with white matter hyperintensities (WMH) and 105 healthy controls), which are high-contrast and noise-free images selected by our clinicians. All *en face* images were acquired at different imaging depths using RTVue XR Avanti SD-OCT system with AngioVue software (Optovue, USA) from the Peking University Third Hospital, Beijing, China. All these images cover a 3 × 3mm^2^ field of view centered at the fovea with 304 × 304 pixels. As the proposed SR-Net requires paired images for training, i.e., clean image and image with stripe noise, in this work stripe noise corrupted image groups were generated by adding stripe noise to those noise-free images in the clean group. In order to obtain the realistic stripe noise added to the noise-free images, we adopted CUD method described in Wu et al. (18) to remove real stripe artifacts from AS-OCTA images, and then extracted the stripe noise images by substracting the corresponding destriping results of CUD from the original stripe noise corrupted AS-OCTA images. Furthermore, we defines the number of stripe noise images added to one noise-free OCTA image as the stripe noise level of the synthetic stripe noise corrupted image. In this work, we synthesized stripe noise images with four stripe noise levels (*i* = 1, 2, 3, 4) to validate destriping capability of the proposed method for different noise levels. Figures 5A, C show a pair of clean and its synthetic corruption image at noise level *i* = 2, and Figure 5B illustrates the corresponding stripe noise. In our implementation, 180 pairs images were used for training and the rest for testing.

**Figure 6:**
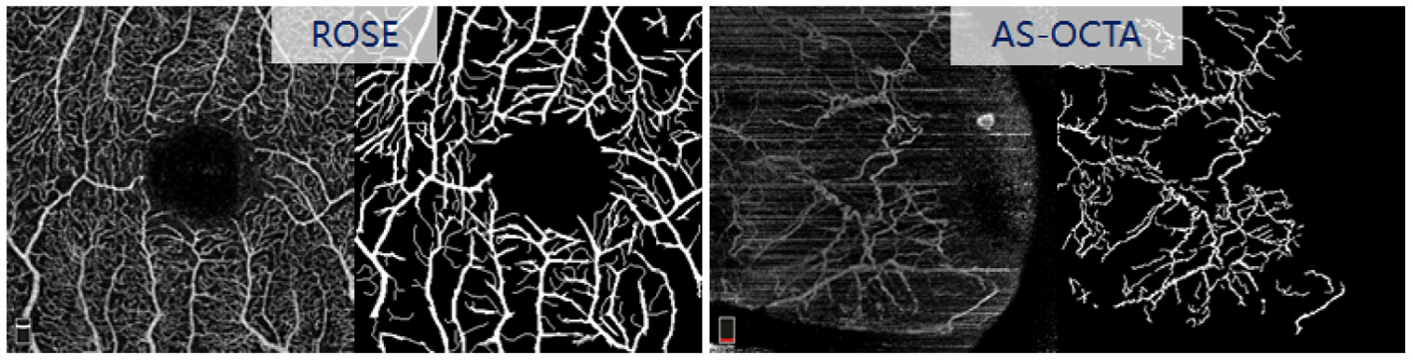
Original OCTA images and their vessel manual annotations in **ROSE** and **AS-OCTA** dataset.

**ROSE** is a recently released OCTA dataset contains 229 images with manual vessel annotations provided, as shown in Figure 6. In this work, we selected the subset ROSE-1 for evaluation. ROSE-1 has 117 images, which were captured by RTVue XR Avanti SD-OCT system with AngioVue software (Optovue, USA), and image resolution is 304 × 304 pixels. We randomly selected 10 images from ROSE-1 and added stripe noise in the same way as above.

**AS-OCTA** is an in-house dataset, which has 31 conjunctival OCTA images collected from the Peking University Third Hospital, Beijing, China. These images were acquired by RTVue XR Avanti SD-OCT system with AngioVue software (Optovue, USA), with a scan area of 6 × 6mm^2^. All the images in this dataset contain different levels of stripe noise, and the reference vessel were manually annotated by an image analysis expert using the open source software ImageJ at pixel-level (see Figure 6).

### 4.2. Evaluation metrics

To verify the effectiveness of our method for stripe noise removal and overall image quality enhancement, two validation strategies were employed. First, we utilized the widely-used image quality assessment metrics, peak signal-to-noise ratio (PSNR) and structural similarity (SSIM), to validate the proposed de-striping network. PSNR is defined as the ratio between power of maximum signal intensity and noise in an image and measures the image fidelity (the larger the better). SSIM measures the similarity of structural information between an image and its high-quality reference version (the larger the better). These two validation approaches were only applied on the datasets with corrupted (synthetic stripe noise added) image, i.e., PUTH and ROSE dataset.

Second, as both the ROSE and our AS-OCTA dataset have vessel annotations, we performed vessel segmentation on the enhanced images to confirm the relative benefits of the proposed framework and the other enhancement methods. Dice coefficient (Dice), Sensitivity (Sen), and G-mean score (G-mean) metrics were employed to evaluate the segmentation performance. These metrics are defined as follows:

(10) $$\begin{array}{l} Dice=\frac{2\times TP}{2\times TP+FP+FN}, \end{array}$$

(11) $$\begin{array}{l} Sen=\frac{TP}{TP+FN}, \end{array}$$

(12) $$\begin{array}{l} G\text{-}Mean=\sqrt{Sen\times Spe}, \end{array}$$

where *Spe* = *FP*/(*FP* + *TN*) indicates the specificity; TP, TN, FP and FN represent the number of true positives, true negatives, false positives and false negatives, respectively.

### 4.3. Implementation details

The proposed framework was implemented using PyTorch library on the PC contained an NVIDIA GeForce GTX 3090 with 24 GB of memory.

#### 4.3.1. SR-Net

We used the training set (*n* = 180) from the PUTH to train our SR-Net. The Adam optimizer with recommended parameters was used to optimize the model and batch size was set as 16. The initial learning rate was 2 × 10^−4^ and gradually decayed to zero after 300 epochs. The hyper-parameters used in the network were λ = 0.002, α = 0.5, β = 2 and γ = 1.

#### 4.3.2. PS-GAN

Due to the lack of high-quality AS-OCTA images for training, and the high similarity of contrast and structural distribution between AS-OCTA image and corneal confocal microscopy, in this work, we combined the training set (*n* = 180) of the PUTH and a corneal confocal microscopy dataset (CCM) to form a new dataset to train our model. The public-accessible dataset CORN-2 (20) was used, and it contains unpaired 340 low-quality and 288 high-quality CCM images. In the experiment, domain *X* consists of low-contrast CCM, and all the images in PUTH and the high-quality images in CORN-2 were included in domain *Y*. All the images in both domains were resized to 400 × 400. The proposed PS-GAN was optimized using Adam and batch size was set to 1. The initial learning rate was 2 × 10^−4^ for the first 100 epochs and gradually decayed to zero after the next 100 epochs. The hyper-parameters used in the network were ξ = 0.5, ρ_1_ = ρ_2_ = 0.5.

## 5. Experimental results

We validated individual components of our two-stage OCTA image quality improvement framework: stripe noise removal and image enhancement stages.

### 5.1. Validation of SR-Net in stripe noise removal

#### 5.1.1. Visual comparisons

**Figure 7:**
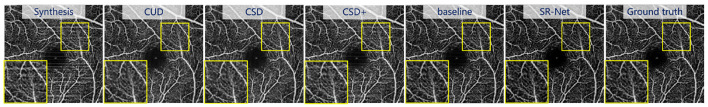
Illustrative stripe removal results using different methods on a synthetic OCTA image (*i* = 2) with stripe noise from the **PUTH** dataset.

**Figure 8:**
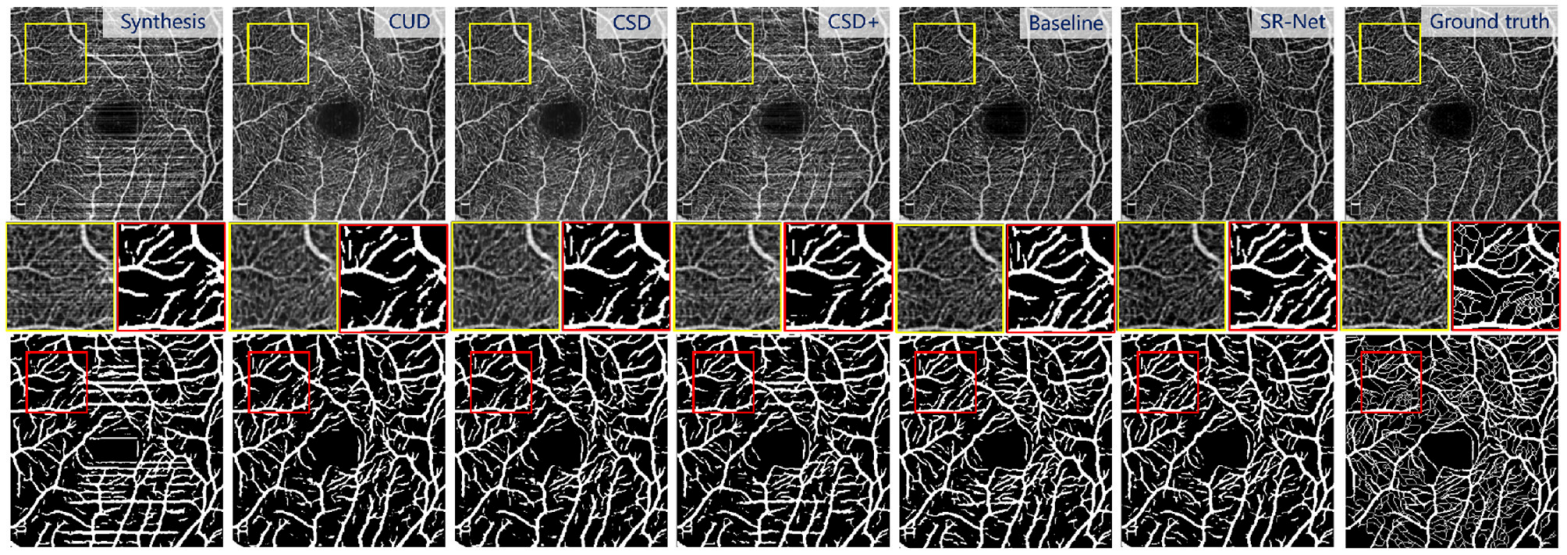
Illustrative results using different stripe removal methods on a synthetic OCTA image (*i* = 2) in **ROSE** dataset, and their vessel segmentation performances by OCTA-Net (4). **Top**: De-striped OCTA images by different methods. **Bottom**: vessel segmentation results. **Middle**: representative patches of de-striped and vessel segmentation results.

As we mentioned in the dataset section, synthetic stripes were added to the images in the PUTH and ROSE datasets. In order to demonstrate the superiority of the de-striping stage, we compared our SR-Net with the state-of-the-art de-striping methods including CUD, CSD and CSD+ (18), as well as the baseline model (U-Net). Figures 7, 8, top present the stripe noise removal results produced by the different methods. As illustrated in Figure 7, top, SR-Net removes stripe noise more faithfully without losing vessel information comparing with other methods. CSD+ attempts to remove the effect of stripe artifacts from the given images, but it still contains noticeable stripe artifacts. Overall, the proposed SR-Net generate the best performance in eliminating stripe noise, i.e., with more visually informative results.

#### 5.1.2. Evaluation by PSNR and SSIM

It is difficult to demonstrate conclusively the superiority of the enhancement method purely by the above visual inspection, in this section, a quantitative evaluation of de-striping is provided. The PSNR and SSIM values of different methods on both the PUTH and ROSE datasets are shown in the Table 1. As we can see, the higher stripe noise level (*i* = 1, 2, 3, 4, the larger *i*, the stronger corruption) usually lead to the lower PSNR and SSIM. It is evident that our SR-Net achieves the highest PSNR and SSIM under various levels of noise corruption. For those OCTA images with low-level stripe noise, all de-striping methods could achieve relatively high performance, and the proposed SR-Net achieves a slightly higher PSNR and SSIM scores than other approaches. For example, SR-Net achieves the improvements of only 8.98, 8.86, 9.16, 1.08, 1.08, and 0.21% when, respectively, compared with CUD, CSD, and CSD+ in terms of PSNR and SSIM on ROSE dataset at noise level *i* = 2. By contrast, the performance of CUD, CSD and CSD+ declines and the proposed SR-Net yields better performance with relatively more significant margins for those OCTA images with high-level stripe noise. For example, the proposed SR-Net achieves an increase of about 31.72, 31.71, 36.63, 7.01, 7.01, and 9.11% in terms of PSNR and SSIM when, respectively, compared with CUD, CSD, and CSD+ at noise level *i* = 4. Similarly, our method also yields better performance with large margins on the PUTH dataset when the images were corrupted by high-level noise. It indicates that the proposed SR-Net is robust to different stripe noise levels.

**Table 1:** De-striping performance in **ROSE** and **PUTH** dataset in terms of PSNR and SSIM.

**Data**	**Noise level**	***i*** = 1		***i*** = 2		***i*** = 3		***i*** = 4	
	**Metrics**	**PSNR**	**SSIM**	**PSNR**	**SSIM**	**PSNR**	**SSIM**	**PSNR**	**SSIM**
PUTH	Synthesis	25.679 ± 1.212	0.938 ± 0.019	24.422 ± 1.799	0.898 ± 0.030	21.854 ± 1.303	0.855 ± 0.032	19.844 ± 1.141	0.813 ± 0.040
	CUD	28.568 ± 0.795	0.942 ± 0.008	26.119 ± 0.910	0.924 ± 0.013	23.959 ± 0.894	0.904 ± 0.017	21.982 ± 0.984	0.883 ± 0.021
	CSD	28.609 ± 0.807	0.942 ± 0.008	26.149 ± 0.918	0.924 ± 0.013	23.972 ± 0.899	0.905 ± 0.017	21.986 ± 0.984	0.884 ± 0.021
	CSD+	26.786 ± 0.988	0.955 ± 0.011	26.047 ± 1.543	0.930 ± 0.019	23.393 ± 1.163	0.899 ± 0.021	21.224 ± 1.080	0.866 ± 0.029
	Baseline	30.746 ± 0.511	0.955 ± 0.004	28.211 ± 0.469	0.942 ± 0.004	27.702 ± 0.577	0.935 ± 0.005	27.309 ± 0.607	0.928 ± 0.004
	SR-Net	**31.608 ± 0.446**	**0.960 ± 0.008**	**28.907 ± 0.475**	**0.948 ± 0.009**	**29.117 ± 0.519**	**0.949 ± 0.008**	**29.371 ± 0.512**	**0.950 ± 0.007**
ROSE	Synthesis	24.644 ± 1.570	0.902 ± 0.022	22.528 ± 2.186	0.871 ± 0.040	21.646 ± 1.331	0.851 ± 0.032	19.960 ± 1.109	0.811 ± 0.036
	CUD	25.963 ± 1.397	0.924 ± 0.014	26.427 ± 1.050	0.926 ± 0.011	23.937 ± 1.105	0.903 ± 0.017	22.166 ± 0.889	0.884 ± 0.019
	CSD	25.987 ± 1.412	0.924 ± 0.014	26.457 ± 1.059	0.926 ± 0.011	23.959 ± 1.121	0.903 ± 0.017	22.168 ± 0.896	0.884 ± 0.019
	CSD+	24.895 ± 2.042	0.917 ± 0.025	26.384 ± 1.382	0.934 ± 0.013	23.310 ± 1.262	0.898 ± 0.023	21.370 ± 0.961	0.867 ± 0.026
	Baseline	27.705 ± 0.748	0.928 ± 0.004	28.438 ± 0.647	0.934 ± 0.007	27.640 ± 0.606	0.933 ± 0.008	27.237 ± 0.664	0.926 ± 0.008
	SR-Net	**28.140 ± 0.541**	**0.936 ± 0.014**	**28.801 ± 0.519**	**0.936 ± 0.013**	**29.025 ± 0.432**	**0.945 ± 0.012**	**29.197 ± 0.617**	**0.946 ± 0.012**

The values in bold represent the best of all the comparative experimental results.

#### 5.1.3. Evaluation by vessel segmentation

We also perform vessel segmentation over de-striped images to confirm the relative benefits of the proposed method in comparison to the others. For vessel segmentation in OCTA images, we employed a recent proposed vessel segmentation network which is designed for OCTA images in the ROSE dataset: OCTA-Net (4). We utilized OCTA-Net to perform vessel segmentation on images with synthetic stripe noise and images after applying different de-striping methods.

The Figure 8, bottom shows the vessel segmentation results by different method on a noise corrupted image. The benefit of the proposed SR-Net method for segmentation may be observed from the representative region (red patches). It can be seen that more accurate and completed visible vessels have been identified by our SR-Net compared with original images with stripe noise and other destriping methods despite difficulties in small vessel detection. By contrast, a large portion of stripe noise has been identified as vessels by OCTA-Net in synthetic images and the de-striped images after applying other de-striping methods. This finding is also evidenced by segmentation results reported in Table 2. It is indeed that our SR-Net improves the segmentation performances when compared to the results of synthetic images: by an increase of about 7.79, 3.40, 7.80, and 8.42% in Dice and 2.71, 1.33, 2.96, and 3.59% in G-mean, respectively with various levels of stripe noise added in the original images. Moreover, when compared with CUD method, SR-Net achieves improvement of about 1.06, 0.79, 1.06, and 1.33% in Dice, 0.72, 0.72, 0.72, and 1.21% in G-mean, respectively, with various levels of stripe noise added. When compared with CSD method, SR-Net achieves improvement of about 1.06, 0.93, 1.06, and 1.33% in Dice, 0.60, 0.84, 0.72, and 1.09% in G-mean with the corruption of different noise levels. Similar to the results of PSNR and SSIM, vessel segmentation performance gain with larger margin has also been achieved by the proposed SR-Net when the images were corrupted by high-level noise.

**Table 2:** Vessel Segmentation on de-striped images in **ROSE** dataset, in the presence of different levels of synthetic stripe noise.

**Methods**	**Different levels of synthetic stripe noise**											
	*i* = 1			*i* = 2			*i* = 3			*i* = 4		
	**Dice**	**Sen**	**G-mean**	**Dice**	**Sen**	**G-mean**	**Dice**	**Sen**	**G-mean**	**Dice**	**Sen**	**G-mean**
Synthesis	0.706 ± 0.027	0.718 ± 0.035	0.813 ± 0.024	0.736 ± 0.039	0.725 ± 0.035	0.825 ± 0.027	0.705 ± 0.040	0.713 ± 0.030	0.810 ± 0.027	0.701 ± 0.027	0.705 ± 0.034	0.807 ± 0.023
CUD	0.753 ± 0.038	0.725 ± 0.037	0.829 ± 0.029	0.755 ± 0.040	0.725 ± 0.039	0.830 ± 0.029	0.752 ± 0.040	0.722 ± 0.041	0.828 ± 0.031	0.750 ± 0.041	0.720 ± 0.034	0.826 ± 0.028
CSD	0.753 ± 0.038	0.725 ± 0.041	0.830 ± 0.058	0.754 ± 0.040	0.723 ± 0.037	0.829 ± 0.029	0.752 ± 0.040	0.723 ± 0.041	0.828 ± 0.030	0.750 ± 0.042	0.721 ± 0.031	0.827 ± 0.027
CSD+	0.741 ± 0.030	0.721 ± 0.031	0.825 ± 0.025	0.754 ± 0.039	0.727 ± 0.032	0.831 ± 0.027	0.742 ± 0.038	0.722 ± 0.032	0.825 ± 0.027	0.739 ± 0.040	0.718 ± 0.038	0.823 ± 0.030
Baseline	0.757 ± 0.034	0.731 ± 0.031	0.833 ± 0.025	0.760 ± 0.037	0.733 ± 0.034	0.835 ± 0.027	0.756 ± 0.035	0.733 ± 0.033	0.834 ± 0.026	0.756 ± 0.037	0.728 ± 0.033	0.831 ± 0.026
SR-Net	**0.761 ± 0.034**	**0.734 ± 0.030**	**0.835 ± 0.024**	**0.761 ± 0.035**	**0.738 ± 0.029**	**0.836 ± 0.024**	**0.760 ± 0.035**	**0.733 ± 0.029**	**0.834 ± 0.024**	**0.760 ± 0.036**	**0.736 ± 0.037**	**0.836 ± 0.028**

The values in bold represent the best of all the comparative experimental results.

For vessel segmentation in AS-OCTA dataset, we employed the Curvelet denoising based Optimally Oriented flux enhancement method (COOF) (61), which was proposed for segmentation of retinal microvasculature in OCTA images. It is worth noting that, on one hand, no pre-trained deep learning models may be utilized to performance the segmentation in AS-OCTA dataset, due to the lack of high-quality AS-OCTA images. On the other hand, it is our purpose to validate the proposed de-striping method would benefit both deep learning-based and conventional segmentation methods.

**Figure 9:**
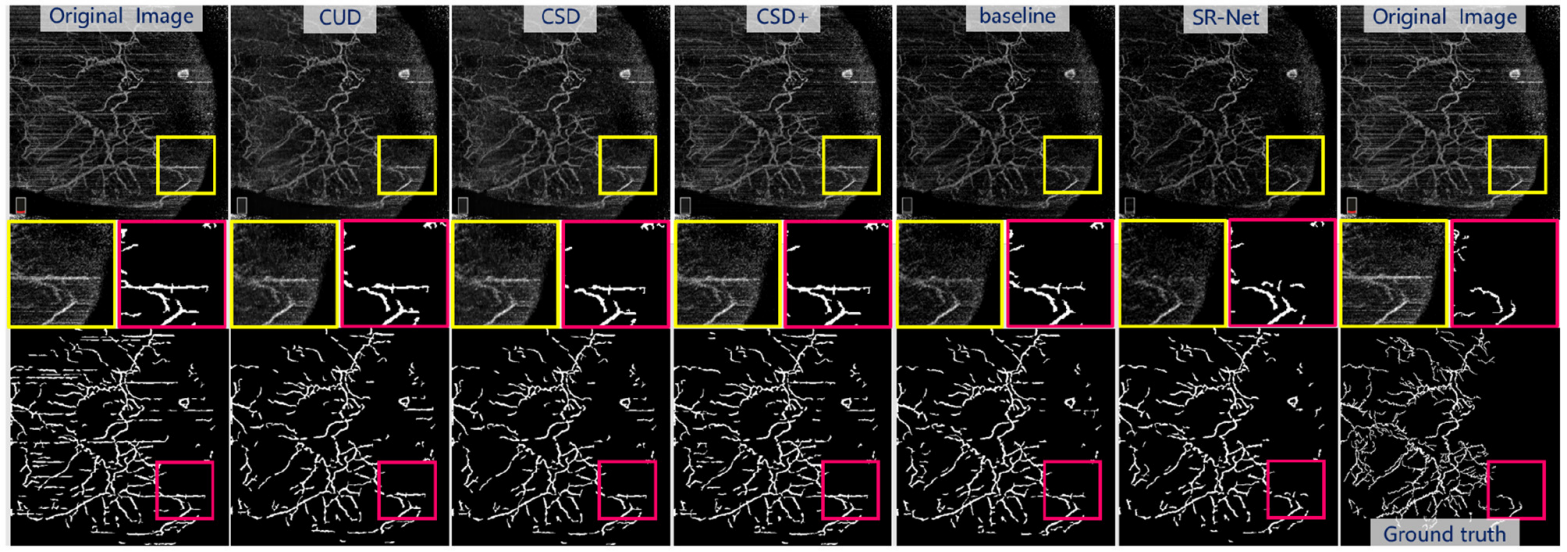
Illustrative stripe removal results using different methods on a real **AS-OCTA** image and their vessel segmentation performance by COOF (61). **Top:** De-striped AS-OCTA images by different methods. **Bottom:** Vessel segmentation results. **Middle:** Representative patches of de-striping and vessel segmentation results.

As shown in Figure 9, bottom and the representative patches (red box), it may be seen clearly that COOF is able to identify vessel structures more accurately in the images with less stripe noise falsely detected as vessels after our SR-Net is applied. We can observe that the competing methods have retained some stripe noise in Figure 9, top and the representative patches (yellow box), and the appearance of stripe and vessels are of great similarity, thus the COOF method falsely detects stripe noise as vessels. By contrast, most stripe noise similar to vessels has been well removed by the proposed SR-Net. Furthermore, **Table 4** demonstrates the superiority of the proposed SR-Net in improving segmentation performances. It shows that our method yields the highest scores in terms of Dice, Sen, and G-mean. SR-Net achieves G-mean of 0.714, with an improvement of about 5.78%, 5.62%, and 3.03% over CUD, CSD, and CSD+.

In summary, the proposed SR-Net is helpful in improving the accuracy of vessel segmentation, since more stripe noise has been removed, and it would enhance the visibility of the vascular structure for subsequent processing.

### 5.2. Validation of PS-GAN in image enhancement

#### 5.2.1. Visual comparisons

In order to demonstrate the superiority of the re-enhancing stage, we compared our PS-GAN with several state-of-the-art image enhancement methods, including CycleGAN (59), EnlightenGAN (56), CycleDehaze (62), and StillGAN (20). All these competing methods are based on GAN, which aim to translate an image from low-quality to high-quality domain in this work.

**Figure 10:**
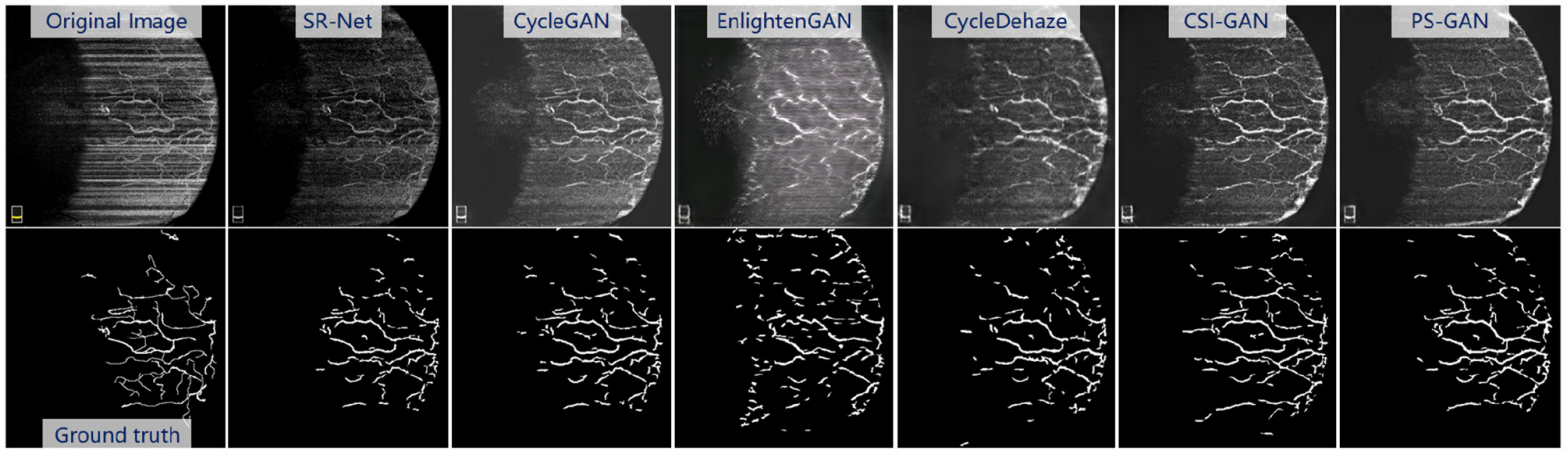
Results of the re-enhancing stage with PS-GAN on an **AS-OCTA** image. The **(top)** are the re-enhancing results after SR-Net and the **(bottom)** are the segmentation results by COOF (61).

The Figure 10, top presents the visual enhancement results *via* different methods. The results obtained by EnlightenGAN and CycleDehaze have image distortion and blurring affect because these methods are hard to guarantee the preservation of details in the images. CycleGAN and StillGAN produced relative better results than EnlightenGAN and CycleDehaze, however, they showed some side effects such as intensity inhomogeneity and vessel discontinuity. In contrast, the proposed method yielded more visually informative results, i.e., relatively visual pleasing contrast and clear visibility of vessel structures.

#### 5.2.2. Evaluation by PSNR and SSIM

We also calculated PSNR and SSIM for quantitative evaluation of re-enhancing stage over the PUTH and ROSE datasets. The quantitative results of different enhancement approaches are shown in Table 3. The proposed PS-GAN obtained the best performances in terms of both metrics - it shows large margin when compared with EnlightenGAN and CycleDehaze as they have changed the content of the given images.

**Table 3:** Comparison of re-Enhancing performance in terms of PSNR and SSIM on the synthetic datasets.

**Data**	**Noise level**	***i*** = 1		***i*** = 2		***i*** = 3		***i*** = 4	
	**Metrics**	**PSNR**	**SSIM**	**PSNR**	**SSIM**	**PSNR**	**SSIM**	**PSNR**	**SSIM**
PUTH	SR-Net	31.608 ± 0.446	0.960 ± 0.008	28.907 ± 0.475	0.948 ± 0.009	29.117 ± 0.519	0.949 ± 0.008	29.371 ± 0.512	0.950 ± 0.007
	EnlightenGAN	12.033 ± 0.456	0.491 ± 0.031	12.025 ± 0.465	0.492 ± 0.031	12.019 ± 0.470	0.493 ± 0.032	12.020 ± 0.465	0.495 ± 0.032
	CycleDehaze	19.611 ± 1.180	0.710 ± 0.046	19.645 ± 1.221	0.713 ± 0.044	19.572 ± 1.131	0.707 ± 0.045	19.572 ± 1.115	0.705 ± 0.044
	StillGAN	29.368 ± 2.416	0.958 ± 0.013	29.088 ± 1.980	0.955 ± 0.012	28.367 ± 2.167	0.949 ± 0.014	27.843 ± 2.063	0.944 ± 0.014
	Baseline	30.186 ± 0.871	0.956 ± 0.004	29.699 ± 0.824	0.951 ± 0.004	29.278 ± 0.710	0.947 ± 0.005	28.907 ± 0.719	0.943 ± 0.005
	Baseline+*L*_*p*_	31.407 ± 0.933	0.964 ± 0.004	30.730 ± 1.001	0.960 ± 0.003	30.190 ± 0.777	0.955 ± 0.004	29.689 ± 0.768	0.951 ± 0.005
	Baseline+*L*_*s*_	31.143 ± 0.669	0.963 ± 0.005	30.469 ± 0.583	0.958 ± 0.005	30.127 ± 0.524	0.955 ± 0.005	29.704 ± 0.507	0.952 ± 0.006
	PS-GAN	**31.736 ± 0.920**	**0.966 ± 0.004**	**30.948 ± 0.904**	**0.961 ± 0.004**	**30.562 ± 0.690**	**0.958 ± 0.005**	**30.014 ± 0.642**	**0.953 ± 0.004**
ROSE	SR-Net	28.140 ± 0.541	0.936 ± 0.014	28.801 ± 0.519	0.936 ± 0.013	29.025 ± 0.432	0.945 ± 0.012	29.197 ± 0.617	0.946 ± 0.012
	EnlightenGAN	11.854 ± 0.309	0.476 ± 0.021	11.886 ± 0.312	0.480 ± 0.021	11.868 ± 0.324	0.481 ± 0.022	11.852 ± 0.334	0.480 ± 0.024
	CycleDehaze	19.916 ± 0.492	0.733 ± 0.023	19.957 ± 0.490	0.735 ± 0.025	19.862 ± 0.394	0.731 ± 0.025	19.761 ± 0.464	0.726 ± 0.027
	StillGAN	29.271 ± 2.560	0.954 ± 0.017	28.882 ± 2.369	0.952 ± 0.017	29.052 ± 2.002	0.951 ± 0.014	28.194 ± 1.835	0.945 ± 0.014
	Baseline	29.457 ± 1.120	0.951 ± 0.007	29.355 ± 1.115	0.950 ± 0.006	29.004 ± 0.968	0.946 ± 0.007	28.564 ± 1.008	0.941 ± 0.007
	Baseline+*L*_*p*_	30.463 ± 1.480	0.957 ± 0.008	30.334 ± 1.359	0.957 ± 0.007	29.727 ± 1.343	0.953 ± 0.009	29.329 ± 1.395	0.948 ± 0.010
	Baseline+*L*_*s*_	30.166 ± 0.907	0.955 ± 0.006	30.250 ± 0.790	0.955 ± 0.006	29.805 ± 0.630	0.952 ± 0.006	29.451 ± 0.654	0.948 ± 0.007
	PS-GAN	**30.810 ± 0.981**	**0.960 ± 0.006**	**30.765 ± 0.946**	**0.960 ± 0.005**	**30.125 ± 0.918**	**0.956 ± 0.006**	**29.647 ± 0.926**	**0.951 ± 0.007**

The values in bold represent the best of all the comparative experimental results.

For example, PS-GAN achieves higher PSNR and SSIM scores in ROSE dataset when compared with EnlightenGAN, CycleDehaze and StillGAN if we added different levels of stripe noise, with 158.83, 54.16, 6.52, 100, 30.61, and 0.84% higher when compared with competing methods in terms of PSNR and SSIM when noise level *i* = 2. Similarly, our method also yields better performance with large margin in the PUTH dataset when the images were corrupted by different noise levels. These findings also demonstrate the robustness of PS-GAN to different stripe noise levels.

#### 5.2.3. Evaluation by vessel segmentation

In order to confirm the impact of the re-enhancing stage on vessel segmentation, we further performed vessel segmentation and compared segmentation results from the enhanced images obtained by different methods on the AS-OCTA dataset.

For the vessel segmentation in the AS-OCTA dataset, we also employed COOF (61) as the vessel segmentation method. The Figure 10, bottom presents the segmentation results *via* COOF, it may be seen that COOF is able to identify vessel structures more accurately on the images after our PS-GAN is applied. Because of the hard presentation of details in the images, the segmentation results of EnlightenGAN and CycleDehaze are deviated from the ground truth. CycleGAN and StillGAN produced relative better segmentation results than EnlightenGAN and CycleDehaze, however, they showed some side effects such as intensity inhomogeneity and vessel discontinuity of vessel segmentation results. In contrast, the proposed PS-GAN yielded more visually informative results, COOF detects more vessel structures. Furthermore, **Table 5** demonstrate the obvious advantage of the proposed PS-GAN in improving Dice, Sen and G-mean of vessel segmentation compared with other competing approaches. Moreover, our PS-GAN achieves G-mean of 0.812, with an improvement of about 12.62%, 13.57% and 8.12% over EnlightenGAN, CycleDehaze and StillGAN, respectively.

### 5.3. Ablation studies

#### 5.3.1. SR-Net

In order to verify the effectiveness of our proposed method for the stripe noise removal, we regarded the U-Net which shares the same structure with SR-Net and adopts mean square error (MSE) loss function as the baseline model and confirmed the stripe removal effectiveness of the proposed loss function *L*_*destripe*_.

*Visually* For synthetic OCTA images in PUTH and ROSE datasets, comparative results of the baseline and our SR-Net are illustrated in Figure 7 and the top row of Figure 8, indicating that the baseline model still contains some noticeable stripe artifacts whilst our SR-Net can remove stripe artifacts satisfactorily. As illustrated in Figure 9, top in AS-OCTA, our SR-Net removes stripe artifacts more cleanly than the baseline, in addition, our method will not lose vessel information.

*Vessel segmentation* For synthesis OCTA with annotations in the ROSE dataset, the vessel segmentation results of baseline and SR-Net are shown in Figure 8, bottom, our method preserves vascular integrity better. This finding is also evidenced by the segmentation results reported in Table 2. When compared with the baseline, the proposed SR-Net achieves improvement of about 0.53, 0.13, 0.53, and 0.53% in Dice, 0.24, 0.12, 0.00, and 0.60% in G-mean with the corruption of different noise levels. For the AS-OCTA dataset, as illustrated in Figure 9, bottom, our SR-Net will remove stripe artifacts more cleanly without loss of any vessel information than the baseline. Furthermore, Table 4 demonstrates the superiority of the proposed SR-Net in improving segmentation performances. The proposed SR-Net achieves an improvement of about 5.88, 1.89, and 1.71% in Dice, Sen, G-mean over the baseline, respectively.

**Table 4:** Vessel segmentation performance on original images in **AS-OCTA** dataset and their de-striped versions *via* different methods.

**Methods**	**Dice**	**Sen**	**G-mean**
Original	0.442 ± 0.161	0.496 ± 0.142	0.529 ± 0.146
CUD	0.499 ± 0.168	0.485 ± 0.165	0.675 ± 0.135
CSD	0.499 ± 0.168	0.485 ± 0.165	0.676 ± 0.135
CSD+	0.489 ± 0.159	0.511 ± 0.158	0.693 ± 0.122
Baseline	0.527 ± 0.174	0.528 ± 0.163	0.702 ± 0.125
SR-Net	**0.558 ± 0.166**	**0.538 ± 0.166**	**0.714 ± 0.132**

The values in bold represent the best of all the comparative experimental results.

#### 5.3.2. PS-GAN

In order to verify the further quality improvement on AS-OCTA images in the re-enhancing stage, we regarded the results de-striped by our SR-Net as the input low-quality imagesof all re-enhancing approaches for unified comparison. CycleGAN was adopted as the baseline and cyclic perceptual loss *L*_*p*_ and structure loss *L*_*s*_ were added to baseline, respectively to confirm the effectiveness of the both.

*Visually* As shown in Figure 10, top, our method will improve the continuity of vessel compared with the baseline, and our method will not generate the non-existent vessels.

*Vessel segmentation* Illustrated in the Table 5, we also applied COOF (61) for the vessel segmentation to verify the effectiveness of enhancement results. Our method achieves the highest Dice, Sen and G-mean when compared with the baseline, Baseline+*L*_*p*_ and Baseline+*L*_*s*_, indicating that the proposed PS-GAN is effective to restore more vascular information. Furthermore, as illustrated in Table 5, the proposed PS-GAN can improve the segmentation performances. The proposed PS-GAN achieves Dice of 0.599, with the improvement of about 0.50, 0.84, and 0.50% over the baseline, The baseline+*L*_*p*_ and baseline+*L*_*s*_. Meanwhile, the proposed PS-GAN achieves G-mean of 0.812, with the improvement of about 1.75, 2.27, and 1.37% over the baseline, baseline+*L*_*p*_ and baseline+*L*_*s*_.

**Table 5:** Vessel segmentation performance on original images in **AS-OCTA** dataset, and their de-striping results of SR-Net and re-enhanced versions *via* different methods, and ablation study of our model.

**Methods**	**Dice**	**Sen**	**G-mean**
Original	0.442 ± 0.161	0.496 ± 0.142	0.529 ± 0.146
SR-Net	0.558 ± 0.166	0.538 ± 0.166	0.714 ± 0.132
EnlightenGAN	0.467 ± 0.102	0.547 ± 0.078	0.721 ± 0.050
CycleDehaze	0.502 ± 0.114	0.531 ± 0.101	0.715 ± 0.071
StillGAN	0.568 ± 0.149	0.592 ± 0.156	0.751 ± 0.108
Baseline	0.596 ± 0.144	0.665 ± 0.122	0.798 ± 0.077
Baseline+*L*_*p*_	0.594 ± 0.140	0.656 ± 0.116	0.794 ± 0.071
Baseline+*L*_*s*_	0.596 ± 0.144	0.673 ± 0.130	0.801 ± 0.078
PS-GAN	**0.599 ± 0.142**	**0.693 ± 0.126**	**0.812 ± 0.073**

The values in bold represent the best of all the comparative experimental results.

In addition, we also validated the effectiveness of *L*_*p*_ and *L*_*s*_ on the synthetic OCTA datasets. As shown in Table 3, PSNR and SSIM were improved by adding *L*_*p*_ or *L*_*s*_ respectively. For example, the proposed SR-Net achieves the improvement in PSNR and SSIM scores in the ROSE dataset when compared with the baseline, baseline+*L*_*p*_ and baseline+*L*_*s*_ if we added different levels of stripe noise, i.e., 3.86, 1.34, 1.07, 1.06, 0.31, and 0.42% higher in terms of PSNR and SSIM for noise level *i* = 3. Similarly, our method also yields better performance in the PUTH dataset when the images were corrupted by different noise levels. Furthermore, the proposed PS-GAN achieves the best performance on image enhancement compared with the baseline, baseline+*L*_*p*_ and baseline+*L*_*s*_.

## 6. Discussions and conclusions

In medical imaging, it often has two main degradation factors: imaging noise and poor contrast. The existing enhancement methods usually address contrast adjustment and noise reduction separately. In this paper, we have proposed a novel two stage framework that is effective across a variety of medical imaging modalities, in addressing noise, and poor contrast simultaneously. To this end, we introduced a stripe loss, perceptual loss, and structure loss to constrain the information of the stripe distribution, contrast and vessel structures respectively, in OCTA images.

In the de-striping stage, an encoder-decoder architecture with stripe noise constraints called SR-Net is proposed to remove stripe noise in AS-OCTA and synthesized OCTA images. In the re-enhancing stage, in order to obtain better quality images, a PS-GAN is developed to translate a de-stripped image from low-contrast domain to high-contrast domain *via* the cross-modality training strategy.

Our method has advantages of simple implementation, high efficiency and wide applicability, e.g., OCTA and AS-OCTA. The effectiveness of this enhancement framework was validated by conventional image quality assessment metrics and the application of vessel segmentation. Experimental results confirm that the proposed method can remove stripe artifacts and achieve higher quality than other state-of-the-art methods either in public or in-house datasets. In addition, the vessel segmentation performance showed that the proposed method yields a promising enhancement performance, that enables both conventional and deep learning-based segmentation methods to produce improved segmentation results across two OCT image modalities.

We believe that our work is of the interest of the computer vision community. Firstly, our task focuses on image enhancement and denoising, which would find applications in the other areas of object modeling, classification and recognition. Secondly, although we present our work based on medical image data, our algorithm is not medical-specific. The proposed loss functions are generalizable and can be used to enhance the performance of other networks such as U-Net and GAN. In the future, we will involve the research on explanation of the models and discuss what the proposed framework focuses on during the enhancement processing, such as regions with stripe noise or low contrast. In addition, we will consider combining stripe artifacts removal and image enhancement into an end-to-end process. Furthermore, This work can also be extended to other OCTA devices such as Zeiss and Heidelberg systems and we will further improve stability and robustness of the model on multi-center OCTA data. The potential application of the proposed framework is even not limited in the medical image domain, and can be applied in many other computer vision tasks, e.g., scratch detection for industrial quality assurance and enhancement of remote sensing images.

## Data availability statement

The original contributions presented in the study are included in the article/supplementary material, further inquiries can be directed to the corresponding author.

## Ethics statement

The studies involving human participants were reviewed and approved by Ningbo Institute of Materials Technology and Engineering, Chinese Academy of Sciences. The patients/participants provided their written informed consent to participate in this study.

## Author contributions

JC and ZX conceptualized the topic, researched and analyzed the background literature, wrote the manuscript, and including interpretations. MX, YM, and YZ provided substantial scholarly guidance on the conception of the topic, manuscript draft and interpretation, and revised the manuscript critically for intellectual content. All the authors approved the final version of the manuscript, ensured the accuracy and integrity of the work, and agreed to be accountable for all aspects of the work.
